# Interactions in Ternary Aqueous Solutions of NMA and Osmolytes—PARAFAC Decomposition of FTIR Spectra Series

**DOI:** 10.3390/ijms222111684

**Published:** 2021-10-28

**Authors:** Emilia Kaczkowska, Aneta Panuszko, Piotr Bruździak

**Affiliations:** Department of Physical Chemistry, Gdańsk University of Technology, Narutowicza 11/12, 80-233 Gdańsk, Poland; ekaczkowska@poczta.onet.pl (E.K.); aneta.panuszko@pg.edu.pl (A.P.)

**Keywords:** PARAFAC, FTIR spectroscopy, osmolytes, N-methylacetamide, weak interactions

## Abstract

Intermolecular interactions in aqueous solutions are crucial for virtually all processes in living cells. ATR-FTIR spectroscopy is a technique that allows changes caused by many types of such interactions to be registered; however, binary solutions are sometimes difficult to solve in these terms, while ternary solutions are even more difficult. Here, we present a method of data pretreatment that facilitates the use of the Parallel Factor Analysis (PARAFAC) decomposition of ternary solution spectra into parts that are easier to analyze. Systems of the NMA–water–osmolyte-type were used to test the method and to elucidate information on the interactions between *N*-Methylacetamide (NMA, a simple peptide model) with stabilizing (trimethylamine *N*-oxide, glycine, glycine betaine) and destabilizing osmolytes (n-butylurea and tetramethylurea). Systems that contain stabilizers change their vibrational structure to a lesser extent than those with denaturants. Changes in the latter are strong and can be related to the formation of direct NMA–destabilizer interactions.

## 1. Introduction

The thermodynamic equilibrium between the folded and unfolded states of proteins in aqueous solutions can be affected by the addition of small organic molecules called osmolytes. Osmolytes can be divided into two groups depending on how they modulate protein stability: stabilizers (e.g., trimethylamine *N*-oxide, glycine, and betaine), which shift the equilibrium towards the native state, and destabilizers (e.g., urea and its alkyl derivatives), which favor the unfolded state of the protein [1,2,3]. Despite many studies, it is still not possible to present a clear view on the exact mechanism of action of osmolytes. Two general mechanisms have been proposed: direct and indirect; both are not mutually exclusive. According to the direct mechanism, denaturants interact directly with the protein, while stabilizing osmolytes are preferentially excluded from the protein surface, which results in its preferential hydration [4,5,6,7,8]. The indirect mechanism assumes that osmolytes affect the protein by modifying the properties of the surrounding water [9,10,11,12,13]. Understanding the mechanism of the influence of osmolytes on protein stability is critical to the proper functioning of proteins and to elucidate the protein folding and unfolding mechanism.

Due to the complex nature of the interactions in protein–osmolyte–water solutions, simple model systems are used. *N*-Methylacetamide (NMA) is considered as a minimal part of the peptide backbone that contains a peptide linkage and, additionally, two hydrophobic methyl groups. Aqueous solutions of NMA in the presence of osmolytes have been studied mainly theoretically [14,15,16,17,18,19] and rarely experimentally [20,21,22], and they have provided information about various interactions that play a role in the protein folding process.

Chemometric methods of the isolation of the spectra of a mixture components are widely used in spectroscopic studies [23,24,25,26,27,28,29,30,31]. However, three-way methods of spectral analysis (such as Parallel Factor Analysis (PARAFAC)) [32,33] are rarely employed in studies using FTIR spectroscopy [34,35]. In such cases, rather, two-way chemometric methods in the analysis of such spectra series are used. Moreover, most of these methods require some data modification (rotation), so that the isolated spectra of the mixture components have a close to physical meaning (MCR, PCA, PFA, etc.). Such a modification of raw data is not perfect and may introduce some artifacts into the results [36,37]. Three-way spectra series (e.g., spectrofluorimetric) are measured with an additional data “axis”, and this additional “dimension” imposes constraints on the spectral isolation method. The results of the chemometric isolation of spectra from such three-way data reflect the real spectral composition. Additionally, some hidden factors can be elucidated, i.e., those that carry information on intermolecular interactions in the case of our studies.

The aim of this study was to chemometrically isolate FTIR spectra, which would provide direct information on potential direct or indirect interactions in NMA–water–osmolyte systems. A whole series of spectra contains two main factors: spectra of pure mixture components and hidden spectra corresponding to changes caused by interactions. The spectra of pure components are known. However, the spectral changes caused by the interactions are not known in advance and are the most important in the study of interactions in ternary mixtures.

The experimental preparation of a solution series that would allow the use of the three-way method of spectra isolation turned out to be easier than expected, yet more laborious. It is in fact similar to that proposed by Noda in his work concerning 2D-FTIR spectroscopy [38,39] or the difference spectra method developed by Stangret [9,40]. Both of these methods rely on the introduction of external change (here, mainly a change in solute concentration) to a system (i.e., a mixture of a solvent and and/or two solutes). The drawback is that both methods provide information on spectral changes caused only by one affecting factor (i.e., change in concentration, temperature, etc.). However, we propose a method of spectral data preparation that allows us to isolate spectral changes caused by two external factors at the same time (here, changes in concentrations of NMA and an osmolyte) with the discrimination power of a three-way chemometric method, PARAFAC. The potential time gain of factor isolation with PARAFAC is greater than the time involved in manual analysis leading to similar results. The presented scheme of sample preparation and pretreatment of spectral series is designed to obtain the most reliable spectra possible with the least possible contribution of the human factor at the analysis stage.

## 2. Results and Discussion

### 2.1. Stabilizers: TMAO, GLY, and BET

**Figure 1:**
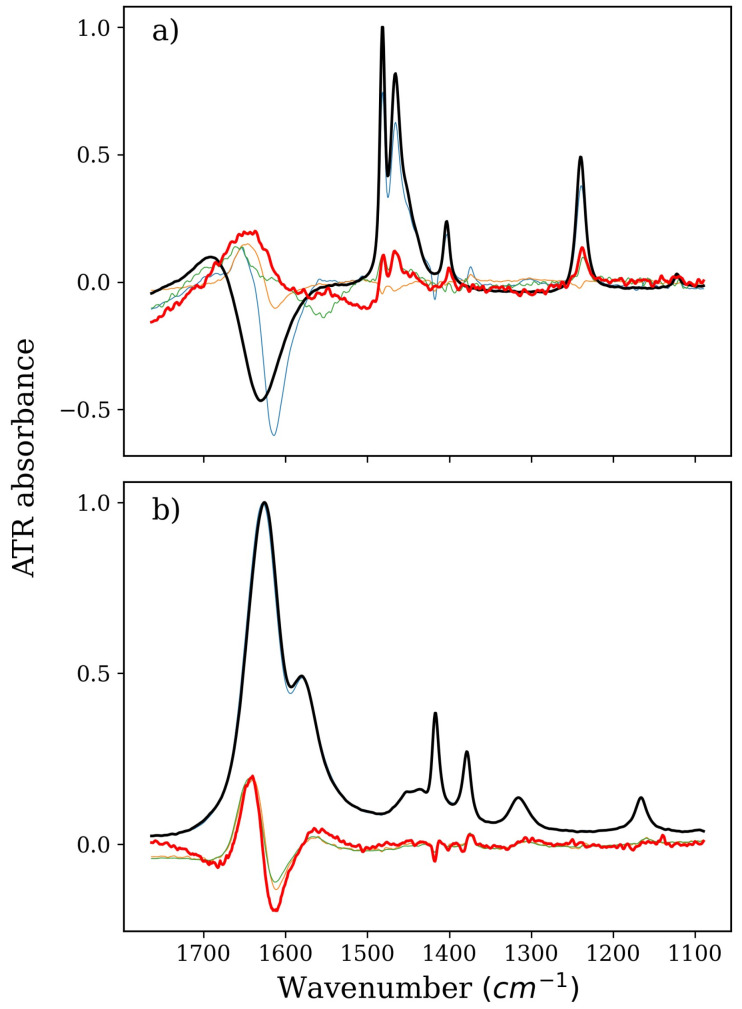
Spectra of pure TMAO or NMA solution (thick black lines), derivatives, i.e., spectral representation of changes in pure solutions of TMAO or NMA caused solely by their concentration increase (thick red lines), and isolated spectra of factors from a series devoid of (**a**) water and NMA or (**b**) water and TMAO (all other thin lines). Part (**a**), therefore, denotes TMAO, and Part (**b**) denotes NMA, both corresponding to the green and magenta series in Figure 6b, respectively. Absorbance is arbitrary; spectra intensities were adjusted to fit into one figure.

TMAO is a unique stabilizer. It strongly enhances the structure of water, despite having only one significant center of interaction with water molecules [12,41,42,43]. Its influence on water is also clearly visible on the spectra of the factors obtained from the decomposition of the isolated TMAO series (Figure 1). TMAO itself does not have bands in the amide I region on FTIR spectra (1700–1600 cm^–1^), but the very change in its concentration is expressed by a disturbance of the OH bending region of water (ca. 1700–1600 cm^–1^). This change, of course, can only come from changes in the vibrational structure of water molecules in the environment of the TMAO. In the presence of NMA in the solution, changes in this range are even stronger and become difficult to analyze. The bending OH band of water is not the best one to precisely describe the water structure. Thus, we used other available data to provide information on possible solute–solvent interactions.

**Figure 2:**
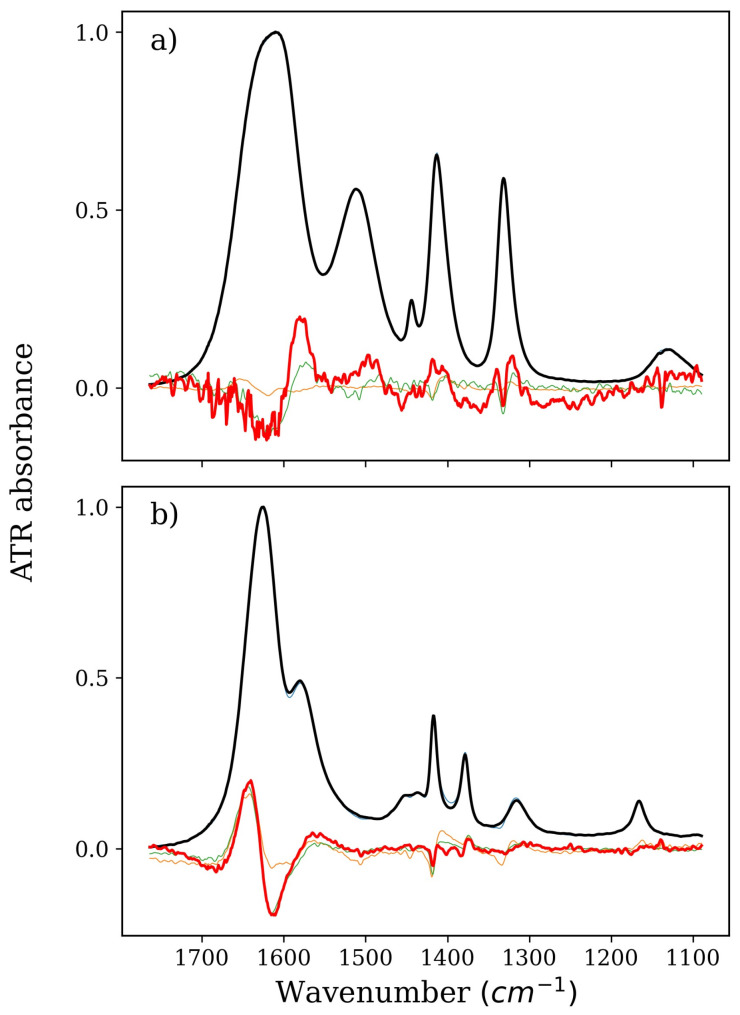
Spectra of pure GLY or NMA solution (thick black lines), derivatives (thick red lines), and isolated spectra of factors from a series devoid of (**a**) water and NMA or (**b**) water and GLY (all other thin lines). Part (**a**), therefore, denotes GLY, and Part (**b**) denotes NMA, both corresponding to the green and magenta series in Figure 6b, respectively.

In the NMA–water–GLY system, the spectra of the factors indicate that the potential interactions are weak and not direct (Figure 2). The additional factor in the series is similar to the derivative spectrum of NMA with an extra band in the range of N–H bending vibrations. The same factor also exhibits a shift of the C–N stretching and C–H deformation bands (ca. 1420 cm^–1^ and 1330 cm^–1^, respectively) towards higher numbers, which suggests an increase in hydrophobic hydration.

**Figure 3:**
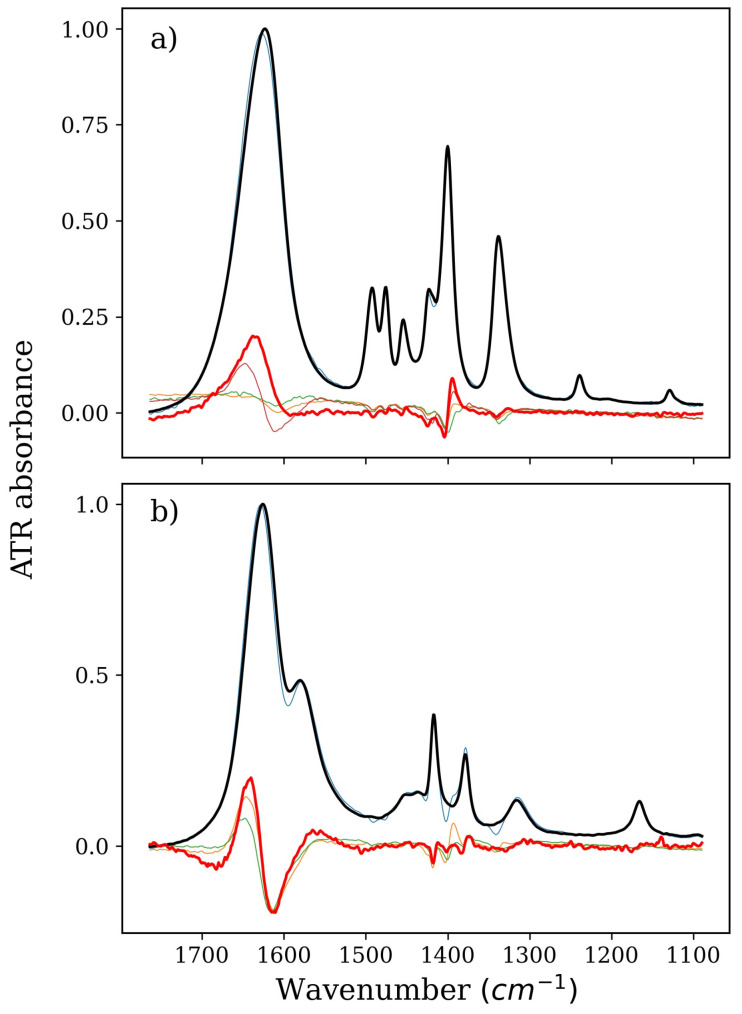
Spectra of pure BET or NMA solution (thick black lines), derivatives (thick red lines), and isolated spectra of factors from a series devoid of (**a**) water and NMA or (**b**) water and BET (all other thin lines). Part (**a**), therefore, denotes BET, and Part (**b**) denotes NMA, both corresponding to the green and magenta series in Figure 6b, respectively.

In the case of BET, apart from the factor that duplicates the changes in the series of NMA factors, there is an additional factor that is clearly different from the BET derivative spectrum (Figure 3). These changes indicate a shift of the amide I band accompanied by a change in its intensity (characteristic wave-like shape). However, it must be stressed that this difference band may be for the amide I (carbonyl stretching group) or bending vibrations of the OH band of water. However, there is no evidence of direct interactions with NMA, as NMA itself virtually shows no changes in the range of the N–H bending band, which is the only significant chemical group that can interact with the betaine carboxyl group. These results indicate the possibility of indirect influences of betaine on the NMA structure. Therefore, it can be concluded that the interactions are mainly localized in the vicinity of the carboxyl group and may indicate the reorganization of water molecules around BET. Additionally, the C–N stretching and C–H bending bands are involved to some extent in this reorganization, but much less than in the GLY system. Changes in the position of these bands are already visible in the BET derivative spectrum (and visible in the NMA factors), and in the presence of NMA, they are slightly inhibited. Since there are no changes characteristic of the shifts of these bands, a cautious conclusion can be drawn that the structure of the BET molecule in the presence of NMA is not altered by the change in concentrations of solutes. This can indicate that the charge distribution throughout the molecule is somewhat displaced. The different behavior of these bonds in relation to the GLY system is certainly directly related to the lack of a direct interaction of the positively charged part of the BET molecule with the surrounding water.

The shapes of the factors of the spectral series of the isolated NMA in the case of all these stabilizers mainly reproduce the derivative spectrum of NMA and pure NMA. In fact, there are only two significant factors in the series. This means that both NMA and the stabilizing osmolyte tend to avoid each other in solution. In the case of the NMA–TMAO system, there can be doubts, but in this case, the distinct variability of one of the factors in the series of isolated NMA can be explained by the large influence of TMAO itself on water. No more nonoverlapping factors were obtained, which additionally confirms this thesis. In the case of GLY, the influence of the system components on water is negligible, so it is much easier to notice that there are no changes in the spectra of the factors in the isolated series of NMA. Both factors are pure NMA and a reconstructed derivative NMA spectrum, respectively. Additional bands at approximately 1400 cm^–1^ are a clear duplication of changes originating from the affected GLY (they are present on the factor in the same place and have the same shape). Similarly, in the case of BET, the changes in the spectra of the factors in the NMA series are very small, and the only discrepancies between the derivative spectrum of NMA in this system and the spectra of the factors can be easily attributed to changes in the vibrational structure of the BET molecule.

### 2.2. Denaturing Agents: TMU and NBU

**Figure 4:**
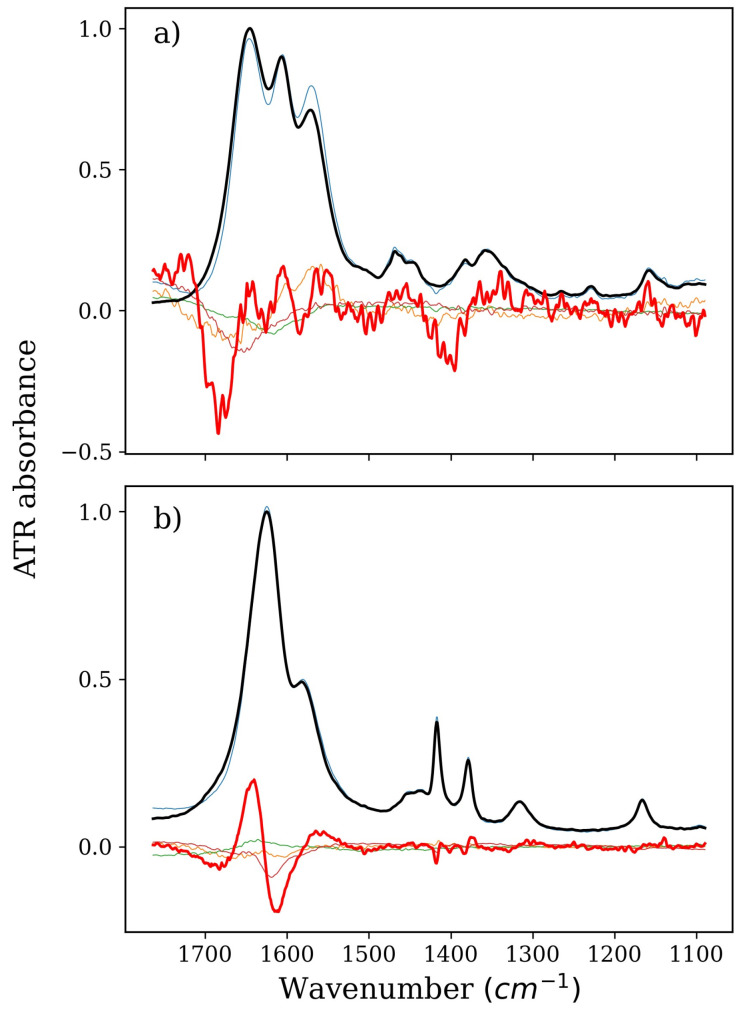
Spectra of pure NBU or NMA solution (thick black lines), derivatives (thick red lines), and isolated spectra of factors from a series devoid of (**a**) water and NMA or (**b**) water and NBU (all other thin lines). Part (**a**), therefore, denotes NBU, and Part (**b**) denotes NMA, both corresponding to the green and magenta series in Figure 6b, respectively.

Changes caused by the increase in the concentration of NBU in a pure aqueous solution are weak, as evidenced by a relatively distinct noise in the derivative spectrum of NBU solutions (Figure 4). This spectrum is not reproduced as one of the factors from the series of spectra of NMA–NBU solutions, which only strengthens the theory of direct interactions. This also means that NBU does not behave in the presence of NMA in the same way as it does in its pure solution. Large changes in the shape of the factors in the range of the amide bands I and II with the simultaneous lack of changes in the vibrations of the CH bending bands indicate that the hydrophilic fragment of the NBU molecule plays a greater role in the formation of potential direct NMA–NBU interactions (amide bands I and II correspond to the vibrations C=O and N–H and O–H). The large number of factors of various shapes in this series indicates that many different methods of interaction are likely and there is no dominant one. We previously suggested [9] that its hydrophobicity might be the reason why NBU is a strong protein denaturant. Its hydrophobic chain induces relaxation of the protein surface. We confirmed this thesis, yet a small additional remark must be made: the hydrophobic tail of the NBU molecule, after fulfilling its role, i.e., exposing the hydrophobic internal fragments of the protein chain, does not participate in the denaturation mechanism. Rather, it facilitates the formation of direct interactions of the hydrophilic part of NBU with the main chain.

**Figure 5:**
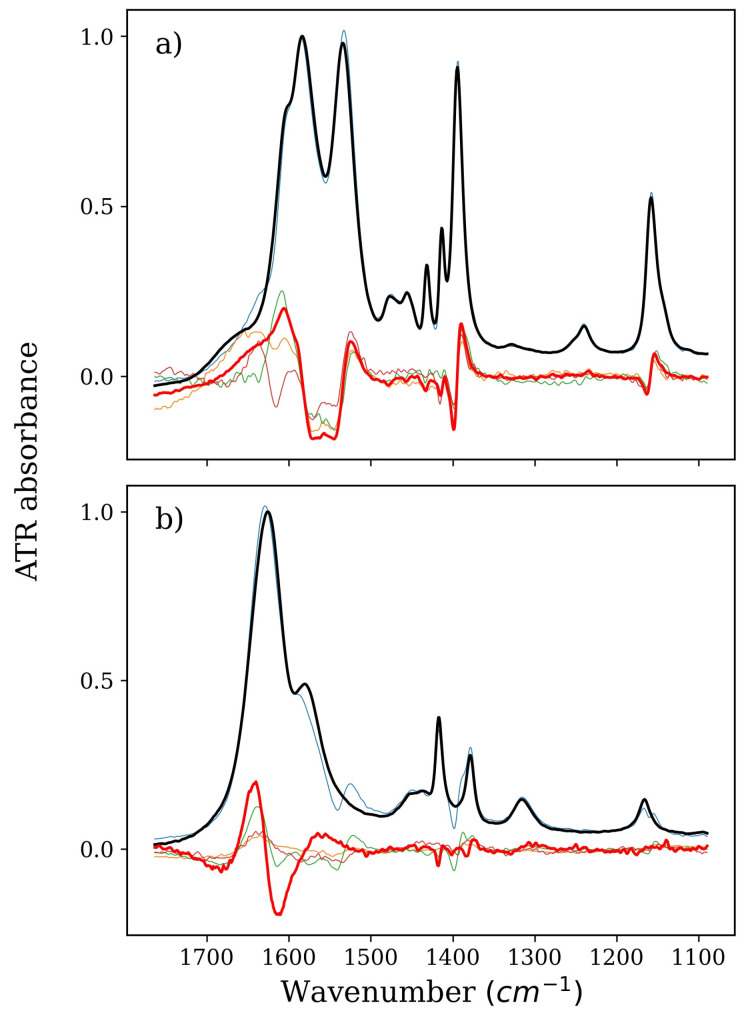
Spectra of pure TMU or NMA solution (thick black lines), derivatives (thick red lines), and isolated spectra of factors from a series devoid of (**a**) water and NMA or (**b**) water and TMU (all other thin lines). Part (**a**), therefore, denotes TMU, and Part (**b**) denotes NMA, both corresponding to the green and magenta series in Figure 6b, respectively.

The derivative spectrum of TMU is much clearer and smoother than in the case of NBU (Figure 5). All the most important types of bonds and groups of bonds of the molecule in its derivative spectrum are shifted. As in the case of the system containing NBU, the derivative spectrum of TMU is not fully reproduced in the system containing TMU and NMA; however, the spectra of the factors isolated from this series are more similar to it than in the previous case, and the shapes of the factors are more convergent. This is not, however, very surprising, as there are many fewer possibilities for the formation of direct interactions between TMU and NMA than in the case of NBU.

Contrary to the aqueous solutions of stabilizers, the factors isolated from the denaturant-based systems lack the one that corresponds to changes in the pure NMA solution, i.e., the one that is similar to the derivative spectrum of NMA. The shapes of the factors are clearly different, which proves different types of interactions for the case of stabilizers and NMA. This suggests a higher probability of direct interactions of two solutes, i.e., TMU or NBU and NMA. It can be said that two NMA molecules in denaturant solutions do not “see” each other in the same way as in stabilizer solutions. In the solutions of the selected denaturants, other types of interactions are forced. Other changes in the spectra of factors isolated from the NMA series are clearly duplicates of changes in the spectra of denaturants.

## 3. Materials and Methods

### 3.1. Chemicals and Solutions

*N*-Methylacetamide (NMA, ≥99%, Aldrich, Darmstadt, Germany), Glycine (GLY, ≥99%, Sigma, Darmstadt, Germany), glycine Betaine (BET, ≥99%, Alfa Aesar, Karlsruhe, Germany), Trimethylamine *N*-Oxide (TMAO, ≥99%, Fluka, Steinheim, Germany), *N,N,N’,N’*-Trimethylurea (TMU, 99%, Aldrich, Darmstadt, Germany), n-Butylurea (NBU, ≥99%, Fluka, Steinheim, Germany), and deionized water (<0.01 S·cm^−1^) were used as supplied.

### 3.2. ATR-FTIR Spectroscopy

Spectra of all prepared solutions were recorded on the Nicolet 8700 FTIR spectrometer (Thermo Scientific, Waltham, MA) equipped with a Ge crystal ATR accessory (6 internal reflections, Specac Ltd., Orpington, Great Britain), the EverGlo IR source, KBr beamsplitter, and DTGS TEC detector. Temperature was maintained at 25.0 ± 0.1 °C using an electronic temperature controller (Specac Ltd., Orpington, Great Britain). Each recorded spectrum was obtained by measuring and averaging 256 independent scans with 2 cm^–1^ resolution. The spectrometer was purged with dry nitrogen to minimize the influence of water vapor and carbon dioxide. The remaining vapor contribution was subtracted using the algorithm developed recently in our laboratory [44]. All ATR-FTIR spectra were analyzed using OMNIC (Thermo Scientific, Waltham, MA, USA) and Python scripts.

### 3.3. Spectra Pretreatment

**Figure 6:**
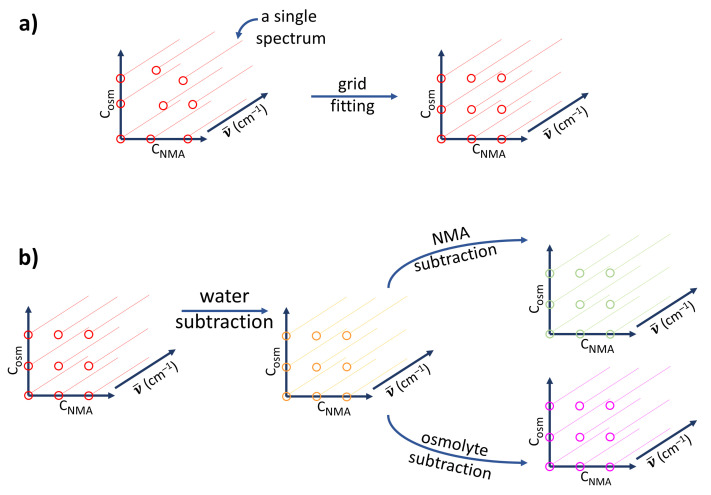
Preprocessing of the spectral series of ternary systems in which the variables are the wavenumber, NMA concentration, and osmolyte concentration. Single spectra are symbolized by colored lines starting with a circle (the wavenumber is arbitrary): (**a**) interpolation to a grid of points in the C_NMA_–C_osm_ plane for each wavenumber (red); (**b**) interpolated spectra after water spectrum subtraction (yellow) are then separated into an NMA-free (green, after NMA spectra subtraction) or osmolyte-free (magenta, after osmolyte spectra subtraction) three-way series. All subtraction factors are calculated from the molar concentrations of the respective solutes or solvent.

Each experimental system consisted of NMA, water, and one of the other organic compounds. For each system, 36 solutions (in a 6 by 6 grid) were prepared with all combinations of concentrations of NMA and the other solute in the range of 0–2 mol·dm^–3^. Later, for each solution, an ATR-FTIR spectrum was recorded. Molar concentrations of NMA and the other solute could be treated as axes of the composition space with pure water in its origin (Figure 6). However, the inevitable experimental error, connected to the stage of solution preparation, meant that those solutions were not strictly evenly spaced (left part of Figure 6a). Such a small randomness of concentrations could be a problem in the further chemometric analysis; thus, for all wavenumbers of ATR-FTIR spectra, planes were first fit to experimental data points and evenly spaced new ones were interpolated (Figure 6a). New data points corresponded to a smaller set of 25 solutions (5 by 5) with evenly spaced concentrations of both solutes. These new concentrations were smaller than in the original set to prevent the instability of data point extrapolation. The method of solution preparation and data interpolation was virtually the same as in our previous paper [45].

The interpolated set of spectra was composed of a few spectral factors, of which three could be directly indicated: pure water, pure NMA in solution, and pure other solute in solution. The extra factors corresponding to possible interactions were obscured by these three factors. Thus, the known factors had to be first subtracted from the 5 by 5 interpolated grid of spectra. As in our previous paper [45], subtraction coefficients based on the molar concentrations of each solution component were calculated and used to first subtract the contribution of pure water from each set of spectra. Such a set of spectra devoid of water’s contribution (yellow symbols in Figure 6b) was next devoid of pure NMA’s or the other solute’s contribution, resulting in a set of the solute or NMA isolated spectra. In this way, two new sets of spectra were created, each composed of mainly NMA or the other solute contributions (green and magenta symbols in Figure 6). Additional bands or shifts corresponding to changes in the vibrational structure of solutes and the solvent were uncovered and easier to analyze.

Two points must be noted. First, the subtraction of water was not complete as it is inevitably affected by the presence of other solutes and can be different from the pure water component. Thus, such a subtraction can be too strict and, in effect, result in the formation of difference bands. However, these bands are valuable because they carry information on possible interactions reflected in changes in intensity or shifts in the IR bands’ positions. Second, some bands in the resulting spectra after all subtractions can be negative. Such negative bands indicate shifts, changes in bandwidth, etc. Thus, some variants of strict non-negative chemometric methods of spectra decomposition cannot be applied. It must also be stressed that both sets of spectra after such a two-step subtraction can carry the same differential information, regardless of the main component of the set. Thus, the analysis of additional or differential bands must be performed each time in relation to the pure spectra of all solution components, including water as the solvent.

### 3.4. Spectra Isolation with PARAFAC

The presented method of experimental design and spectra treatment allowed us to perform PARAFAC analysis of the main spectral components or factors contributing to the overall shape of spectra in the set. In such a three-way dataset, three axes can be indicated: wavenumbers, NMA molar concentration, and the molar concentration of the other solute. The analysis was performed in the Python 3.6 environment with the TensorLy library (v.0.6.0) [46]. As mentioned earlier, the usage of the non-negative PARAFAC was not allowed as negative or differential bands were expected as the result of factor isolation.

The additional dimension of spectral data (here, the additional axis of concentration) imposed constraints during the step of factor isolation, in contrast to two-way factor analysis (or PCA), and resulted in more reliable factors with physical meaning. The only disadvantages of the method of spectra isolation were: (1) the need to interpolate spectral data to an evenly spaced concentration grid; (2) the problem with the determination of the number of real factors. The first one is only of an experimental nature and, in some cases, can be even favorable as it can diminish to some extent experimental errors and smooth the data. The second problem does not have a precise solution, and various ways to determine the number of factors are available in the literature [47]. However, in the case of IR spectra, the number can be estimated on the basis of the shape of isolated factors. We propose the following steps to determine the number: (1) start the decomposition with just two factors; (2) follow with three, then four, etc., and each time, compare the results of decomposition with the results of the previous step; (3) stop when no additional spectral features appear in the factors. The number of factors is determined in the step where new features are still present, and any additional factor does not provide any new information. We were also able to predetermine the minimal number of factors. As in each isolated set, only one main spectral component was present (either NMA or the other solute), we could predict that one factor corresponding to the unaffected component, the second one to the component affected by the change in its own concentration. The third, fourth, etc., factors were the ones that carried the most valuable information on the type and strength of interactions.

We also determined that when the number of factors used for the decomposition was too high, the spectral shape of some of them was almost the same, or some bands were present in more than one factor. Such a redundancy was an indicator that the optimal number of factors was obtained in the previous step.

### 3.5. Derivative Spectra of Solutes

As mentioned above, one of the factors present in the set could be ascribed solely to changes in the solution concentration of a particular solute. This factor cannot be mistaken for the sought-after interaction factors. The identification of such a factor could be conducted by the analysis of changes in the spectral series of pure solute in water (devoid of water spectral contribution). However, for better visualization of the changes caused in such a series, a simple difference can be calculated or, as in this case, a derivative of molar absorbance vs. the solute’s concentration for each available wavenumber. The derivative calculated for a series where only one solute and water are present in various proportions has a spectrum-like shape and emphasizes changes in the band shape of such a spectral series. It is difficult to fully describe this type of spectrum each time it is mentioned; thus, we refer to such spectra as “derivative of NMA” (or TMAO, TMU, etc.) later in the text. However, we literally mean changes in the system due to the concentration of a solute represented as a spectrum.

## 4. Conclusions

We provided an experimental proof that stabilizing molecules (TMAO, GLY, and BET) affect NMA to a lesser extent than denaturing agents (TMU and NBU). The isolation of factors of pure stabilizers or NMA in their solutions clearly indicates that both solutes behave similarly to their pure solutions. GLY can be indicated as the extreme case where factors corresponding to pure GLY and NMA were reproduced perfectly. The TMAO–NMA system is similar: all bands corresponding to solutes were reproduced very well, and only the difference band ascribed to water affected in this system was shifted to lower wavenumbers. Discrepancies in the BET–NMA system were visible, but not significant. On the other hand, the lack of pure denaturant factor in TMU, NBU, and NMA systems supports the idea that this kind of solute is prone to interactions with NMA. Despite the fact that the reproduced factor corresponding to the pure NMA was similar to the pure NMA spectrum (with minor changes easily ascribed to other solutes), no TMU or NBU factors were reproduced as effectively as in the case of stabilizers. The obtained results indicated the formation of direct interactions between destabilizing osmolytes and NMA and no such interaction in the case of stabilizing osmolytes. Accordingly, our results support the indirect mechanism of the effect of the stabilizers on the protein and the direct mechanism of the destabilization, but we do not rule out the interference of these osmolytes in the protein hydration spheres.

It was possible to distinguish, to some extent, both the role of the solvent in the isolation of stabilizer osmolyte molecules from the main protein chain model, i.e., NMA, and differences in the ability to create direct interactions between this peptide model and the stabilizer and denaturant molecules. The chemometric method of choice—PARAFAC—turned out to be a valuable tool to study interactions in ternary solutions.

## Data Availability

All the obtained information are available per request.

## Abbreviations

2D-FTIR	Two-Dimensional Infrared Spectroscopy
ATR	Attenuated Total Reflectance
BET	Glycine Betaine
FTIR	Fourier Transform Infrared Spectroscopy
GLY	Glycine
NBU	n-Butylurea
NMA	*N*-Methylacetamide
PARAFAC	Parallel Factor Analysis
TMAO	Trimethylamine *N*-Oxide
TMU	*N,N,N’,N’*-Trimethylurea
